# Mindfulness-Based Program for Children with Autism Spectrum Disorder and Their Parents: Direct and Long-Term Improvements

**DOI:** 10.1007/s12671-017-0815-x

**Published:** 2017-10-06

**Authors:** Anna Ridderinkhof, Esther I. de Bruin, René Blom, Susan M. Bögels

**Affiliations:** 1 Research Institute of Child Development and Education, University of Amsterdam, Nieuwe Achtergracht 127, 1018 WS Amsterdam, The Netherlands; 2 Karakter, Child and Adolescent Psychiatric Center, Dr. E. Schattenkerkweg 1, 8025 BW Zwolle, The Netherlands; 3 UvA minds, Academic Outpatient Child and Adolescent Treatment Center, University of Amsterdam, Banstraat 29, 1071 JW Amsterdam, The Netherlands

**Keywords:** Autism spectrum disorder, Interventions, Mindfulness, Mindfulness-based program

## Abstract

A combined mindfulness-based program for children and their parents (MYmind) was beneficial for adolescents with autism spectrum disorder (ASD). In this study, we investigated whether this program is also beneficial for younger children with ASD, whether effects last on the long-term, and whether it reduces common comorbid problems. Forty-five children referred with ASD aged 8 until 19 years old, and their parents participated. Repeated measures of children’s and parents’ social communication problems, emotional and behavioral functioning, mindful awareness, and of parenting were conducted pre-intervention, post intervention, 2-month follow-up, and 1-year follow-up. While children did not report significant changes in mindful awareness, their social communication problems decreased, and their emotional and behavioral functioning improved. Results were not consistent at each occasion; improvements reported by children were most substantial at a 2-month follow-up and only partly remained at a 1-year follow-up, while all children’s improvements as reported by parents were present on all occasions. Parents themselves reported improved emotional and behavioral functioning, improved parenting, and increased mindful awareness on all occasions. Parents’ social communication problems reduced only directly after the intervention. Most improvements were supported by the qualitative investigation of children’s and parents’ experienced change as reported on open-ended questions. This study suggests that children, including adolescents, with ASD and their parents can benefit from a mindfulness-based program with parallel sessions for children and parents.

Autism spectrum disorder (ASD) is characterized by difficulties in social communication and interaction, and repetitive and restrictive behavior patterns, interests, or activities (American Psychiatric Association 2013), which affects daily living tremendously. Individuals diagnosed with ASD encounter difficulties in understanding and using nonverbal communication, sharing of emotions, and building social relationships, as well as difficulties with switching in activities and handling changes. Theorized to be underlying the ASD symptoms, several neurocognitive functions are impaired in ASD. Individuals with ASD show overly detailed-focused information processing and weak central coherence (Happé and Frith 2006). In addition, individuals with ASD show difficulties in executive functioning in the domains of planning, cognitive flexibility, inhibition, and working memory (Hill 2004). Furthermore, individuals with ASD show problems with theory of mind, the ability to infer mental states (Baron-Cohen 2000). In addition to these difficulties in information processing, individuals with ASD often show atypical sensory processing (De la Marche et al. 2012).

The global population prevalence of ASD was recently estimated to be 1 in 132 people (Baxter et al. 2015). The prevalence in children is even higher, estimated to be 2.6% in a population-based sample of 7- to 12-year-old children (Kim et al. 2011). ASD is known to be chronic and comes with a lifelong lower level of functioning, quality of life, and need for support (Chiang and Wineman 2014; Seltzer et al. 2004). The utilization and costs of health care services is tremendously higher for children with ASD compared to typically developing children (e.g., Croen et al. 2006; Ganz 2007; Liptak et al. 2006). Costs, including non-health care costs such as children’s school absence or parents’ loss of paid work, are estimated to be 27 times higher for children with ASD and comorbid anxiety disorders than for typically developing children, and four times higher than for children with anxiety disorders (Van Steensel et al. 2013a, b). These figures may indicate the high need for support for individuals with ASD as well as the potential ineffectiveness of the current support.

Raising a child with ASD is highly demanding. Parents of children with ASD show increased parenting stress as compared to parents of typically developing children and to parents of children with other disabilities (e.g., Hayes and Watson 2013; Van Steijn et al. 2014). This leads to parental mental health problems, such as symptoms of depression (Van Steijn et al. 2014). Also, parenting stress and behavior problems of children with ASD increase each other (Bauminger et al. 2010; Hastings 2002). This demanding and stressful situation continues when children with ASD become adult, since parents continue their parental role during adulthood (Seltzer et al. 2004). Together, this points to the importance of intervening effectively during childhood to improve the present and lifelong consequences of ASD for both children and parents, and to reduce the utilization of health care services now and in later life.

Given the difficulties in social communication and neurocognitive functioning, the demands of the social environment are often higher than the coping abilities of children with ASD. According to the transactional stress model, stress occurs when people experience higher demands than their coping abilities, and this leads to physical and psychological stress responses, such as frustration, depression, and anxiety (Lazarus and Folkman 1984). This model may explain why children with ASD, in addition to the core autism symptoms, frequently suffer from emotional and behavioral problems. Children with ASD perceive higher levels of stress and a poorer ability to cope with stress compared to children without ASD (Browning et al. 2009). For 70 % of children with ASD, additional internalizing and externalizing symptoms meet the criteria for at least one comorbid diagnosis (e.g., Simonoff et al. 2008). Anxiety disorders, oppositional defiant disorder (ODD), and attention-deficit/hyperactivity disorder (ADHD) are common comorbid disorders (e.g., Simonoff et al. 2008; Van Steensel et al. 2013a, b). Having comorbid conditions is associated with reduced quality of life in ASD (Chiang and Wineman 2014). Thus, children with ASD are in need for treatment to increase their coping abilities and decrease their stress and comorbid problems.

Although there is a clear need for support, evidence-based treatments for children with ASD are scarce. Several treatment approaches are being examined. According to meta-analyses into early intensive behavioral interventions based on applied behavior analysis, this approach improves adaptive behavior and the intelligence quotient (IQ) (Reichow 2012). However, this intervention approach is studied mainly for children up to 7 years old and consists of an intensive long-lasting program. Treatment gains are largest with one-on-one teaching for at least 20 h per week (Reed et al. 2007), which is not feasible for most families. In addition, long-term outcomes of these intensive programs are still unknown (Matson and Konst 2013). Another treatment approach is social skills training, which seems to improve the skills that are explicitly taught, but fails to generalize to daily life (White et al. 2007). Cognitive behavior therapy for children with ASD is effective in reducing anxiety symptoms and curing anxiety disorders up to 2 years of follow-up (e.g., Storch et al. 2013; Van Steensel and Bögels 2015). However, this treatment is specifically for children with comorbid anxiety disorders and does not target the core autism symptoms or underlying neurocognitive deficits. Medication is used for comorbid behavior problems like impulsivity, anxious mood, or aggressive behavior, but no medical treatment is available for the core symptoms of ASD (Newschaffer et al. 2007). In addition, children with ASD respond equal to placebo as to specific drugs, while adverse effects of these drugs are larger (King et al. 2009). Children have a high sensitivity to adverse effects of medication, such as weight gain or dyskinesia (Almandil et al. 2013; Campbell et al. 1997), so the risks of medication may exceed their benefits. Thus, although several treatment approaches have been investigated, the limited evidence, side effects, lack of generalization, or focus on a specific comorbid disorder of these treatments imply a need for further research into new treatment approaches that have the potential to reduce the negative impact of ASD for children and their parents.

A mindfulness-based program may be such an approach. Mindfulness-based programs are based on the Buddhist traditions of mindfulness meditation and adjusted to mental health care based on the Western science of psychology. Participants train to pay attention to the present moment, on purpose and with a non-judgmental, openhearted, and curious attitude (Kabat-Zinn 1994). They train enhanced attention and awareness of experiences such as bodily sensations, feelings, thoughts, and senses. Also, mindfulness practices are taught to cultivate an accepting and compassionate stance toward experiences (Segal et al. 2012).

Mindfulness-based programs might support children with ASD for several reasons. Firstly, the underlying neurocognitive deficits may improve. Central coherence could be improved by mindfulness, because children practice to shift between widening and narrowing their attention. Rather than paying excessive attention to details that attract attention automatically in ASD, participants train to view both internal and external experiences as passing events in a wider field of awareness (Kabat-Zinn 1994; Segal et al. 2012). Executive functioning could be improved by training mindfulness, because it is practiced to control the focus of attention, to flexibly shift attention, to reflect on experiences, and thereby to notice one’s automatic impulses which enables responding with awareness instead of reacting impulsively (Zelazo and Lyons 2012). For adolescents with ADHD, reduced attention problems and improved executive functioning are found after a mindfulness-based program (Van de Weijer-Bergsma et al. 2012; Zylowska et al. 2008). So, mindfulness training may improve central coherence and executive functioning.

Secondly, this intervention may lead to a reduction in their social communication and interaction problems. Participants cultivate awareness of the present moment, including the interactions with other persons. Being able to pay more attention to social interactions, instead of getting distracted by for example ruminative thoughts or sounds in the surroundings, could help children to be more able to attend to others. In addition, by practicing awareness of one’s own emotions, mindfulness may lead to a better understanding of emotional processes and may thereby improve the understanding of others’ emotions as well. Also, mindfulness may lead to increased awareness of the effect of one’s own behaviors on others (Block-Lerner et al. 2007; Sequeira and Ahmed 2012). Thus, theory of mind, empathy, and social interaction might be improved by mindfulness-based programs.

Thirdly, mindfulness-based programs may improve the coping abilities of children with ASD, by relating less judgmental, more curious, accepting, and compassionate to their experiences, thoughts, and feelings. For example, instead of being caught up in thoughts, trying to understand their feelings, children practice to be aware of the connection between bodily sensations, feelings, and thoughts. They learn to allow them, view them as passing events, and bring their attention back to the present moment. Thereby, this training could reduce their heightened levels of stress, emotional, and behavioral problems. Indeed, mindfulness-based programs reduce stress in healthy adults (Chiesa and Serretti 2009), and reduce stress, anxiety, and depressive symptoms in adults with various psychiatric or medical conditions (Gotink et al. 2015; Hofmann et al. 2010). In addition, in a randomized controlled trial (RCT), a mindfulness-based program was found to reduce psychological inflexibility and mental health problems in adolescents with mixed mental health disorders (Tan and Martin 2014). Furthermore, a mindfulness-based program was found to reduce ADHD symptoms in children and adolescents (Van der Oord et al. 2012; Van de Weijer-Bergsma et al. 2012; Zylowska et al. 2008). Therefore, it is expected that mindfulness-based programs may also reduce stress and the comorbid problems of children with ASD.

Finally, children with ASD and their parents may benefit when parents are trained in mindfulness. Parents of children with ASD experience high levels of stress and consequently suffer from mental health problems (e.g., Hayes and Watson 2013; Van Steijn et al. 2014). Interestingly, higher levels of mindfulness, mindful parenting, acceptance, and self-compassion seem to reduce the impact of children’s behavior problems on parental anxiety, depression, and stress (Jones et al. 2014; Neff and Faso 2015; Weiss et al. 2012). Mindful parenting helps parents to attend in an open, nonjudgmental way to their children, taking their perspectives, and responding calmly instead of reacting automatically (Bögels and Restifo 2014). Thereby, mindful parenting increases their understanding and ability to help their children and themselves. Thus, a mindfulness-based program for parents may intervene in the negative interaction pattern between parental mental health problems and children’s emotional and behavioral problems by increasing parents’ coping abilities. Mindfulness trainings reduce stress, depression, and anxiety in a broad range of populations (e.g., Chiesa and Serretti 2009; Hofmann et al. 2010), thus might also reduce these symptoms in parents of children with ASD. More specifically, a mindful parenting training for parents of children with various mental disorders was found to reduce parenting stress and improve parenting style and co-parenting (Bögels et al. 2014; Meppelink et al. 2016). In addition, a mindfulness-based program was found to be beneficial for adults with ASD (Spek et al. 2013). Heritability in ASD is 80% (Lichtenstein et al. 2010), and ASD symptoms of children and their parents are related (Constantino and Todd 2005). Therefore, a mindfulness-based program for parents of children with ASD may help parents coping with their own ASD symptoms. Together, this indicates that combining a mindfulness-based program for children with ASD with a mindful parenting program could be beneficial for both parents and children.

Few studies investigated the effects of mindfulness-based programs for children with ASD. One small study in children (*n* = 14) with externalizing disorders and their parents included four children with ASD (Bögels et al. 2008). Results indicated improvements on personal goals, internalizing and externalizing symptoms, attention problems, self-control, happiness, and mindful awareness. Furthermore, improvement on sustained attention was found. However, no conclusions could be drawn about the effects for the small subgroup of children with ASD separately. In a series of single-case studies, the effects of a specific mindfulness meditation practice, “Meditation on the Soles of the Feet,” on the reduction of the physical aggression of adolescents with Asperger syndrome (Singh et al. 2011b) and adolescents with autism (Singh et al. 2011a) were investigated. The number of aggressive behaviors was reduced during the training in both studies, with zero aggressive incidents in the last 4 weeks of the mindfulness training and zero to four aggressive incidents in total during a 3- to 4-year follow-up period. Other studies found that a mindfulness-based program of nine weekly sessions for adults with ASD reduced their comorbid symptoms, such as their symptoms of depression, anxiety, rumination, distrust and interpersonal sensitivity, and sleeping problems, and their positive effect increased (Kiep et al. 2015; Spek et al. 2013). Thus, there is emerging evidence for the beneficial effects of mindfulness for children with ASD.

Some studies investigated mindfulness-based programs for the parents of children with ASD. Such programs were found to reduce parenting stress, anxiety, and depression, as well as to improve sleep, global health, well-being, and life-satisfaction of these parents (e.g., Dykens et al. 2014; Ferraioli and Harris 2013; Neece 2014). Moreover, mindfulness training for parents may improve child outcomes as well. A mindfulness-based stress reduction (MBSR) training for parents of children with developmental disabilities (84.8% diagnosed with ASD) was found to decrease children’s comorbid ADHD symptoms compared to waitlist control (Neece 2014). In a series of single-case studies, a mindfulness-based parenting program was found to reduce the adolescents’ aggressive and disruptive behavior and to increase their compliance with the mother’s requests (Singh et al. 2014).

In two pilot studies, mindfulness-based programs for children and their parents were combined. A pilot study explored the effects of a mindfulness training for six mothers of children with ASD, who in turn trained their own children in mindfulness (Hwang et al. 2015). Results indicated an increase in maternal mindfulness and family quality of life, and a reduction in parenting stress and children’s emotional and behavioral problems for most mother-child dyads. Another pilot study investigated a mindfulness-based program for youngsters with ASD (MYmind), combining an adolescent program with a parallel mindful parenting program for their parents (De Bruin et al. 2015). The program was found to be feasible for this population, with an attendance rate of 90%. The 23 adolescents reported a significant increase in their quality of life and reduced rumination, but reported no difference in worry and mindful awareness. Their parents reported an increase in adolescents’ general social responsiveness, including improved social cognition, social communication, and preoccupations. The parents themselves reported an increased mindfulness and improved parenting style with more mindful parenting, less parenting stress, less laxness, and less verbosity. Collectively, these studies provide emerging evidence for the beneficial effects of mindfulness-based programs for children with ASD and their parents.

Although the results from previous studies investigating mindfulness-based programs for children with ASD, their parents, or both are promising, further research is required to investigate whether these preliminary effects can be supported and extended to larger samples, younger age groups, and long-term effectiveness. This study adds to the preliminary findings on mindfulness-based programs for children in combination with mindful parenting training for their parents, by extending the age range to include children from 8 until 23 years old, enlarging the sample size, investigating the effects on comorbid internalizing and externalizing problems, and by adding a 1-year follow-up measurement occasion. Four outcome domains were assessed using a quantitative method, including social communication problems (parents and children), emotional and behavioral functioning (parents and children), parenting (parents only), and mindful awareness (parents and children). We hypothesized improvement on all domains at posttest, 2-month, and 1-year follow-up compared to pre-test. In addition, we explored the subjective experienced changes after the mindfulness-based program using a qualitative method, and evaluated the treatment integrity.

## Method

### Participants

In total, 45 children with ASD (age range 8–19 years) and their parents participated. Table 1 displays the characteristics of the families. All children were included based on their diagnosis of an autistic spectrum disorder according to the Diagnostic and Statistical Manual of Mental Health Disorders guidelines (4th ed., text revised; DSM-IV-TR; American Psychiatric Association 2000). In addition, for 38 (84%) of the children, standardized classification was administered by means of the Autism Diagnostic Observation Schedule (ADOS-G, Lord et al. 2000; ADOS-2, Lord et al. 2012; see Table 1). Children were included if their IQ was estimated to be ≥ 80 based on clinical judgment. For 35.6%, total IQ scores were available from clinical files, and these ranged between 84 and 133 (*M* = 108.1, *SD* = 13.2). Medication for mental problems was used by 17 (38%) children, and 23 (51%) of the families received any additional psychosocial counseling or therapy during the period between the start of the mindfulness training and the booster session (see Table 1 for more detailed information). Exclusion criteria were (1) inadequate mastery of the Dutch language by the parents and/or child, (2) severe behavioral problems as indicated by a conduct disorder, (3) current suicidal risk, and (4) current psychotic disorders.

**Table 1:** Characteristics of participating families

	Children (*n* = 45)	Mothers (*n* = 43)	Fathers (*n* = 31)
Age (mean and SD)	13.03 (2.72)	46.31 (5.22)	46.99 (4.95)
Male	36 (80%)		
ASD diagnosis			
Classic autism	7 (16%)		
Asperger syndrome	14 (31%)		
PPD-NOS	24 (53%)		
Comorbid diagnosis			
ADHD	9 (20%)		
Internalizing disorder^a^	6 (13%)		
ADOS classification			
Autism	11 (24%)		
Autism spectrum disorder	23 (51%)		
One-point beneath cut-off	3 (7%)		
No ASD classification	1 (2%)		
Medication use	17 (38%)		
Additional psychotherapy^b^	23 (51%)		
Parent counseling	8 (18%)		
Family therapy	3 (7%)		
CBT	3 (7%)		
Other therapy for child	4 (9%)		
Combination of therapies	5 (11%)		
Educational type			
Regular education	40 (89%)		
Special education	5 (11%)		
Educational level			
Primary education	22 (49%)		
Secondary education	22 (49%)	11 (26%)	5 (16%)
Vocational training		4 (9%)	4 (13%)
Higher education		18 (42%)	10 (32%)
University	1 (2%)	10 (23%)	11 (35%)
Family situation			
Married/living together	33 (73%)		
Divorced/separated with co-parenting	9 (20%)		
Living with mother	3 (7%)		
Working situation			
Full time		11 (26%)	18 (58%)
Part time		23 (53%)	9 (29%)
Not working^c^		8 (19%)	3 (10%)

*SD* standard deviation, *ASD* autism spectrum disorder, *PPD-NOS* pervasive developmental disorder-not otherwise specified, *ADHD* attention deficit/hyperactivity disorder, *CBT* cognitive behavior therapy

^a^Internalizing disorders included depressive disorder, dysthymic disorder, obsessive compulsive disorder, panic disorder, general anxiety disorder, anxiety disorder-not otherwise specified

^b^Received psychotherapy between the start of the intervention and the booster session

^c^Not working category includes housewife, long-term ill, and unemployed

### Procedure

Families were referred to one of the two participating mental health care centers in the Netherlands, and then asked to participate in the study. Written informed consent was given by parents and by children aged 12 years and older. A repeated measures study design was used with five measurement occasions: waitlist, 1 week pre-intervention, directly post intervention, 2-month follow-up, and 1-year follow-up. The waitlist assessment was conducted 2 months before the mindfulness training started. However, since only six families completed the waitlist, we did not include this measurement occasion in the current study. In addition to repeated measures, parents’ and children’s experienced changes were explored by a qualitative analyses of the evaluation forms. The Medical Ethics Committee of the Academic Medical Center (AMC) in Amsterdam approved the study (NL43720.018.13). This study replicates the pilot study of De Bruin et al. (2015) in a larger participant group with a broader age range, including additional outcome variables and a 1-year follow-up.

### MYmind

The children and one or both parents took part in MYmind, a mindfulness-based program for families with parallel sessions for children and parents. MYmind for families with ASD consists of nine weekly group sessions for children and parents separately, lasting 1.5 h. Children and parents together follow an additional booster session nine weeks after the final session, to encourage continuing with meditation practices. Trainers were child and family mental health care professionals with experience in ASD and had completed an 8-day-long advanced teacher training of MYmind for youth with ADHD/ASD and their parents. Before attending the teacher training, trainers had completed a mindfulness training for themselves (i.e., mindfulness-based cognitive therapy; MBCT), at least one 4-day meditation retreat, and at least a year experience with their own mindfulness practice.

#### MYmind Child Program

The 9-week MYmind child program is a manualized program for youth with ASD, based on the MYmind protocol for youth with ADHD (Van der Oord et al. 2012; Van de Weijer-Bergsma et al. 2012) and on mindfulness-based therapy for adults with ASD (Spek et al. 2013). Children learn to enhance and direct their attention, increase their awareness of bodily sensations, feelings, and thoughts, as well as to increase their self-control. Each session consists of educating theory, practicing mindfulness exercises, inquiry discussions about the exercises, and discussing home practices. The program includes mindfulness exercises based on MBCT (Segal et al. 2012) and MBSR (Kabat-Zinn 1982), such as the breathing meditation, the body scan, the 3-min breathing space, and yoga practices. Home practicing consists of reading handouts, meditation practices, informal mindfulness practices, applying mindfulness practices to daily situations, and diary registrations. The MYmind child program is tailored to the specific problems of children with ASD. For example, more direct language, less metaphors, and a reduced amount of verbal guidance during meditations are used. An additional session is added to allow for more rehearsal. Applying mindfulness in specific situations which are stressful for children with ASD is also incorporated, for example dealing with unexpected changes (e.g., by changing the training room), disturbing sounds (e.g., with a sound meditation), and repetitive thoughts (e.g., with a thought meditation). The purpose and set-up of the inquiry is explained so children know better what to expect. If children encounter difficulties in expressing their experiences when asking open questions during inquiry, two questions are asked (“Was this exercise enjoyable, neutral, or uncomfortable for you?” and “Whereby can this practice help you?”). Children of 8 until 12 years old participated in a different group than adolescents of 12 until 23 years old. Whether a child of 12 took part in the child or adolescent group depended on whether the child was in elementary or high school.

#### MYmind Parent Program

The 9-week MYmind parent program for parents of children with ASD follows an adjusted program based on the Mindful Parenting manual as described in Bögels and Restifo (2014). This manual is a standalone program for parents of children with all kind of disorders and consists of 3-h sessions, while the current MYmind parent program is for a more homogenous group of parents and provided parallel to the children’s sessions of 1.5 h. Therefore, important adaptations were made from the original program. This program includes learning to deal with parenting stress and stressful parenting situations, awareness of automatic reactions toward the child’s behavior and to respond non-automatically, and to respond calmly and open-minded toward the needs of the child and the child’s difficulties. Also, parents learn to be aware of their own boundaries, to bring acceptance and (self-)compassion in their parenting, and to understand and accept the autism of their child. Specific obstacles and needs of parents of children with ASD are used as examples during mindful parenting practices (e.g., a meditation on dealing with frustration of your child that wants to wear a specific set of clothes which are in the washing machine). Home practices consist of reading handouts, meditation practices, informal mindful parenting practices, applying mindfulness practices to daily situations, and diary registrations.

#### Treatment Integrity

To inspect whether MYmind was carried out as intended, we developed the MYmind-Treatment Adherence and Competence Scale (MYmind-TACS) for the child and parent program. In the MYmind-TACS, the adherence to the program and the competence of the trainers were examined. The treatment adherence part examines the degree to which the different components of the MYmind protocol were carried out during each session. The scale ranged from 0 to 2. The treatment competence part examines the degree to which mindfulness trainer skills are visible during each session. The competence scale was based on items of the Mindfulness-Based Interventions Teaching Assessment Criteria (MBI-TAC; Crane et al. 2013), and the Mindfulness-Based Cognitive Therapy Adherence Scale (MBCT-AS; Segal et al. 2002), but simplified to fit the clinical practice of teaching mindfulness to children with ASD and their parents. Items such as “To what extent does the trainer show a curious attitude?”, and “To what extent does the trainer facilitate the group learning process?” were assessed. The scale ranged from 1 to 5.

Video tapes were available for 82 sessions (65%) of the child program and 60 sessions (48%) of the parent program. We trained a research assistant, who had completed her own mindfulness training, in the MYmind-TACS. A second observer (EB) rated 10% of the available videotapes, seven sessions of the child program and seven sessions of parent program videotapes, to evaluate interrater reliability. For the adherence scale, the interrater reliability was excellent (Cicchetti 1994): percentage absolute agreement = 100% and intraclass correlation coefficient (ICC) = 1.00 for both the child and parent program. For the competence scale, a different picture emerged. Although percentage absolute agreement and percentage close agreement were good to excellent (73.5 to 95.9% respectively) for the MYmind child program, the ICC was poor (.27; Cicchetti 1994). This poor ICC is most likely due to a low variation in scores on the competence items, while differentiating between the high scores (4 and 5) appeared difficult. For the MYmind parent program, the percentage absolute agreement was moderate (61.4%), while the percentage close agreement was excellent (95.7%), and the ICC was good (.63; Cicchetti 1994).

Mean adherence score averaged for each session was 1.97 (SD = 0.09) for the MYmind child program and 1.80 (SD = 0.29) for the MYmind parent program. Mean competence score averaged for each session was 4.86 (SD = 0.18) for the child program and 4.63 (SD = 0.26) for the parent program. These scores indicate a high adherence and trainer competence of the intervention in this study.

### Measures

A multi-informant design was used. Children reported about their emotional and behavioral functioning, and mindful awareness. Part of the questionnaires was only completed by children of 11 years and older (*n* = 29), since these questionnaires are not validated in younger children. In addition, parents reported about the children’s social communication problems and emotional and behavioral functioning. Furthermore, parents reported about their own social communication problems, emotional and behavioral functioning, parenting, and mindful awareness. Cronbach’s *α* of each questionnaire reflects the internal consistency on pre-test. The 3-item Self-Compassion Scale Short Form (SCS-SF; Raes et al. 2011) completed by children and the Test Observation Form (TOF; Verhulst and Van der Ende 2013) completed by research assistants were omitted from further analyses because of low reliability (SCS-SF: *α* = .33; TOF: *α* = .57). The teacher-report SRS was omitted because of a low completion rate (max. 11 teachers on one occasion). In addition, to explore the experienced changes qualitatively, parents and children completed an evaluation form with open-ended questions.

#### Children’s Outcomes

##### Social Communication Problems

Parents reported on their children’s social communication problems with the 65-item Social Responsiveness Scale, Dutch version (SRS; Roeyers et al. 2011). This questionnaire inquires about children’s severity of social behavior deficits associated with ASD, including their ability for reciprocal social behavior, communicative deficits, and characteristic autistic preoccupations. Cronbach’s *α* was .94 for both mothers and fathers.

##### Emotional and Behavioral Functioning

To index children’s emotional and behavioral functioning, the broadband syndrome scales internalizing and externalizing problems and the subscale attention problems of the Achenbach System of Empirically Based Assessment, Dutch version (ASEBA; Verhulst and Van der Ende 2013) was used. Parents reported on the Child Behavior Checklist (CBCL) and children 11 years and older reported on the Youth Self Report (YSR). For internalizing problems, Cronbach’s *α* was .90 for the CBCL of mothers, .86 for the CBCL of fathers, and .82 for the YSR. For externalizing problems, Cronbach’s *α* was .91 for the CBCL of mothers, .89 for the CBCL of fathers, and .76 for the YSR. For attention problems, Cronbach’s *α* was .78 for the CBCL of mothers, .78 for the CBCL of fathers, and .70 for the YSR. Furthermore, children 11 years and older completed the 22-item Ruminative Response Scale (RRS; Raes et al. 2003) indexing rumination, which refers to how people think and behave in response to feelings of sadness and depression. Cronbach’s *α* was .95. Children of all ages reported about their perceived stress on the 19-item Chronic Stress Questionnaire for Children and Adolescents (CSQ-CA; De Bruin et al. 2017). Cronbach’s *α* for this questionnaire was .86. Children of all ages completed the 20-item Chronic Sleep Reduction Questionnaire (CSRQ; Meijer 2008). This questionnaire indexes sleep reduction and the consequences of chronic sleep reduction in irritation, energy loss, and sleepiness. Cronbach’s *α* was .82. The World Health Organization-Five Well-Being Index (WHO-5) was completed by children of all ages to index emotional well-being (De Wit et al. 2007). Cronbach’s *α* was .84.

##### Mindful Awareness

Children 11 years and older reported about their trait mindfulness on the 10-item Children’s Acceptance and Mindfulness Measure, Dutch version (CAMM; De Bruin et al. 2014). Cronbach’s *α* was .72.

#### Parents’ Outcomes

##### Social Communication Problems

Parents reported on their own social communication problems with the Dutch version of the 64-item SRS-Adult form (SRS-A; Noens et al. 2012). Cronbach’s *α* was .83 for mothers and .94 for fathers.

##### Emotional and Behavioral Functioning

Parents reported on their internalizing and externalizing problems using the 39-item and 35-item broadband syndrome scales of the Adult Self Report (ASR) of the ASEBA (Verhulst and Van der Ende 2013). Cronbach’s *α* for internalizing problems was .85 for mothers and .88 for fathers, and for externalizing problems, .82 for mothers and .75 for fathers. The 15-item subscale attention problems of the ASR were used to index parents’ attention problems. Cronbach’s *α* was .81 for mothers and .83 for fathers. The stress experienced by parents was assessed by the 14-item Perceived Stress Scale (PSS; Cohen et al. 1983), with Cronbach’s *α* .88 for mothers and .86 for fathers.

##### Parenting

The 13-item competence scale of the Parenting Stress Index, Dutch version (PSI-C; De Brock et al. 1992) was used to assess parents’ stress about their competence in parenting. Cronbach’s *α* was .84 for mothers and .90 for fathers. Parents completed the 5-item overreactivity subscale of the Parenting Scale (PS; Rhoades and O'Leary 2007) to index overreactivity in their parenting style. Cronbach’s *α* was .79 for mothers and .69 for fathers.

##### Mindful Awareness

Parents completed the Dutch 29-item version of the Interpersonal Mindfulness in Parenting Scale (IM-P; De Bruin et al. 2014) to index mindfulness in the parenting relationship. Cronbach’s *α* in this study was .87 for mothers and .86 for fathers. Parents further completed the 3-item SCS-SF (Raes & Neff, personal communication with Raes, based on the 12-item SCS-SF of Raes et al. 2011). Cronbach’s *α* was .72 for mothers and .67 for fathers.

#### Evaluation Form

Parents and children completed a short open-ended questionnaire during the posttest or 2-month follow-up, with questions about: what, if anything, they learned; what, if any, changes they experienced; their opinions about the MYmind program; and what, if anything, has changed in the relationship with their child (for parents only).

### Data Analyses

#### Statistical Analysis

**Fig. 1:**
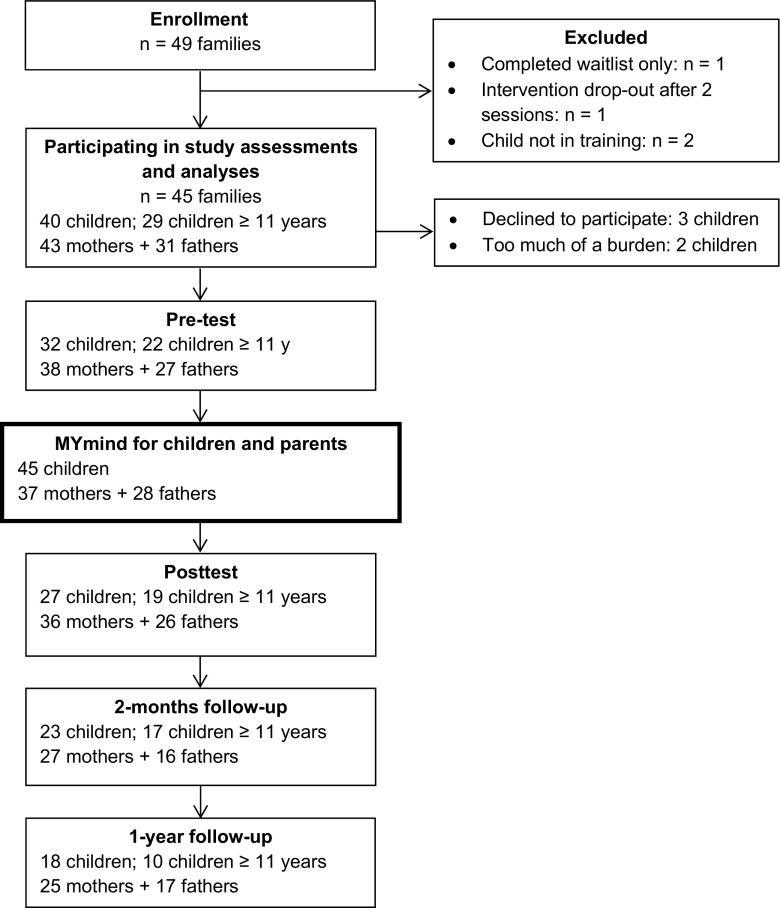
Flow diagram with number of participants at each measurement occasion

We used multilevel analysis to test differences over time on the standardized outcome measures, with measurement occasions nested within participants, and father- and mother-reports about their child nested within children. Thereby, the analysis accounted for possible dependencies within individuals, and all available data could be incorporated. Figure 1 displays the availability of data for each measurement occasion. Inspection of the data revealed five univariate outliers (standardized *z* score < − 3.29 or > 3.29): one on pre-test RRS, one on posttest YSR attention problems, one on posttest PSS, one on posttest SRS-A, and one on 2-month follow-up SRS-A, which were replaced by *z* 3.29. Measurement occasions were included as predictors, with posttest, 2-month, and 1-year follow-up compared to pre-test. In addition, to explore whether differences over time could be explained by children’s age, attendance rate, or additional therapy (additional psychotherapy or medication versus no additional therapy), these variables were included as predictor and in interaction with the measurement occasions in separate analyses for each outcome measure.

#### Qualitative Analysis

Qualitative evaluation data was incorporated into ATLAS.ti and analyzed following the guidelines of conventional content analysis as described by Hsieh and Shannon (2005). The aim of this qualitative part was to explore the changes that participants experienced after the mindfulness-based program. In addition, some parents responded with experienced changes of their children. Therefore, children’s and parents’ answers were analyzed together (by AR). As a first step, all data were read to obtain a sense of the whole. The second step was to read the data word by word and specifying codes linked to quotes that captured key thoughts and concepts. In the third step, notes of first impressions were made. In the fourth step, codes were specified that represented more than one quote or key thought. In the fifth step, codes were organized into categories. In the sixth step, the categories were organized in main- and subcategories using a tree diagram. In the fifth and sixth steps, the original quotes were constantly revisited in a process of constant comparison (Ryan and Bernard 2003), to check if the code indeed fitted the category. In addition, the final 16 categories were verified by a second independent coder. Interrater reliability between the coders was good; *κ* = .74.

## Results

Results of the multilevel models are displayed in Tables 2 and 3. Parameter estimates at posttest and follow-ups represent the deviation from pre-test and can be interpreted as Cohen’s *d* effect sizes—0.20 is considered small, 0.50 medium, 0.80 large (Cohen 1992).

**Table 2:** Parameter estimates for the children’s outcomes social communication problems, emotional and behavioral functioning, and mindful awareness directly after MYmind, at 2-month follow-up, and at 1-year follow-up

	Effects over time			
	Posttest	2-month follow-up	1-year follow-up	Intercept
Social communication problems				
SRS^a^	− 0.32 (0.09)**	− 0.33 (0.10)**	− 0.51 (0.10)**	0.23 (0.13)^
Emotional and behavioral functioning				
CBCL internalizing^a^	− 0.35 (0.11)**	− 0.38 (0.12)**	− 0.63 (0.13)**	0.29 (0.13)*
YSR internalizing^b^	− 0.13 (0.25)	− 0.50 (0.26)^	− 0.59 (0.30)^	0.19 (0.21)
CBCL externalizing^a^	− 0.21 (0.09)*	− 0.43 (0.10)**	− 0.42 (0.10)**	0.30 (0.14)*
YSR externalizing^b^	− 0.20 (0.21)	− 0.56 (0.22)*	− 0.61 (0.26)*	0.32 (0.20)
CBCL attention^a^	− 0.32 (0.10)**	− 0.44 (0.11)**	− 0.58 (0.11)**	0.28 (0.13)*
YSR attention^b^	− 0.22 (0.20)	− 0.57 (0.21)**	− 0.68 (0.24)**	0.42 (0.21)^
RRS^b^	− 0.44 (0.20)*	− 0.71 (0.20)**	− 0.27 (0.25)	0.34 (0.20)
CSQ-CA	− 0.20 (0.17)	− 0.63 (0.18)**	− 0.25 (0.19)	0.21 (0.17)
CSRQ	− 0.06 (0.16)	− 0.28 (0.17)^	− 0.12 (0.18)	0.21 (0.17)
WHO-5	0.35 (0.20)^	0.40 (0.21)^	0.46 (0.23)*	− 0.33 (0.18)^
Mindful awareness				
CAMM^b^	0.02 (0.23)	0.37 (0.24)	0.01 (0.30)	− 0.08 (0.21)

Parameter estimates with standard errors between brackets. Outcomes are standardized so that parameter estimates can be interpreted as Cohen’s *d* effect sizes

*SRS* Social Responsiveness Scale, *YSR* Youth Self Report, *CBCL* Child Behavior Checklist, *RRS* Ruminative Responsiveness Scale, *CSQ-CA* Chronic Stress Questionnaire for Children and Adolescents, *CSRQ* Chronic Sleep Reduction Questionnaire, *WHO-5* World Health Organization-Five Well-Being Index, *CAMM* Children’s Acceptance and Mindfulness

^a^Parent reports about their child

^b^Children from 11 years and older reported on these questionnaires

^*p* < .10, *p < .05, **p ≤ .01

**Table 3:** Parameter estimates for the parents’ outcomes social communication problems, emotional and behavioral functioning, parenting, and mindful awareness directly after MYmind, at 2-month follow-up, and at 1-year follow-up

	Effects over time			
	Posttest	2-month follow-up	1-year follow-up	Intercept
Social communication problems				
SRS-A	− 0.19 (0.08)*	− 0.08 (0.09)	− 0.09 (0.09)	0.15 (0.12)
Emotional and behavioral functioning				
ASR internalizing	− 0.31 (0.10)**	− 0.47 (0.11)**	− 0.37 (0.11)**	0.26 (0.12)*
ASR externalizing	− 0.39 (0.11)**	− 0.51 (0.12)**	− 0.37 (0.12)**	0.35 (0.12)**
ASR attention	− 0.26 (0.10)**	− 0.28 (0.11)*	0.04 (0.11)	0.15 (0.12)
PSS	− 0.43 (0.12)**	− 0.35 (0.14)*	− 0.20 (0.14)	0.26 (0.12)*
Parenting				
PSI-C	− 0.21 (0.10)*	− 0.39 (0.11)**	− 0.28 (0.11)*	0.24 (0.12)^
PS-O	− 0.55 (0.11)**	− 0.65 (0.12)**	− 0.39 (0.12)**	0.41 (0.12)**
Mindful awareness				
IM-P	0.42 (0.12)**	0.51 (0.13)**	0.42 (0.13)**	− 0.37 (0.12)**
SCS-SF	0.28 (0.12)*	0.38 (0.13)**	0.48 (0.13)**	− 0.34 (0.12)**

Parameter estimates with standard errors between brackets. Outcomes are standardized so that parameter estimates can be interpreted as Cohen’s *d* effect sizes

*SRS-A* Social Responsiveness Scale–Adult form, *ASR* Adult Self Report, *PSS* Perceived Stress Scale, *PSI-C* Parenting Stress Index–Competence subscale, *PS-O* Parenting Scale–Overreactivity subscale, *IM-P* Interpersonal Mindfulness in Parenting, *SCS-SF* Self-Compassion Scale Short Form

^*p* < .10, *p < .05, **p ≤ .01

### Children’s Outcomes

#### Social Communication Problems

**Fig. 2:**
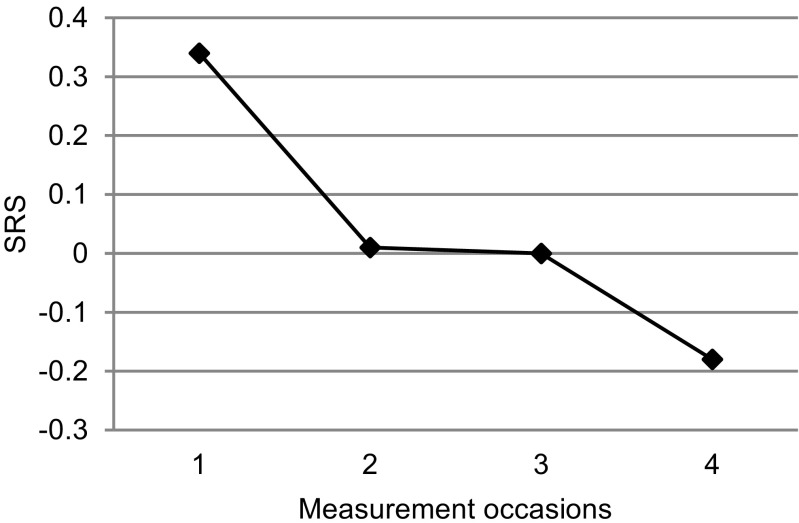
Social communication problems of children over time, as reported by parents on the Social Responsiveness Scale (SRS). Standardized estimated marginal means are calculated based on the parameter estimates that were obtained through the multilevel analysis. *1* pre-test; *2* posttest; *3* 2-month follow-up; *4* 1-year follow-up

Parents reported a significant decrease in children’s social communication problems (SRS) with a small effect from pre-test to posttest. This small decrease was maintained at a 2-month follow-up, and at a 1-year follow-up, a significant decrease with a medium effect size occurred (Fig. 2).

#### Emotional and Behavioral Functioning

Parents reported a significant decrease in children’s internalizing symptoms (CBCL) with a small effect size from pre- to posttest and to 2-month follow-up, and at 1-year follow-up with a medium effect. Internalizing symptoms as reported by children (YSR) were not significantly reduced at posttest, and the medium reduction at 2-month and 1-year follow-up only approached significance. Parents reported a small but significant decrease in children’s externalizing symptoms (CBCL) at posttest, 2-month, and 1-year follow-up. Externalizing symptoms as reported by children (YSR) reduced with a medium and significant effect at 2-month and 1-year follow-up, but the decrease was not significant at posttest. Parents reported a significant decrease in children’s attention problems (CBCL) with a small effect size at posttest and 2-month follow-up, and a medium effect at 1-year follow-up. Attention problems as reported by children (YSR) were not significantly reduced at posttest but were reduced with a medium effect at 2-month and 1-year follow-up. Rumination (RRS) was significantly decreased with a small effect at posttest, and with a medium effect at 2-month follow-up. The decrease in rumination was no longer significant at 1-year follow-up. Children reported no significant reduction in stress (CSQ-CA) at posttest and 1-year follow-up, but did report a significantly reduction at 2-month follow-up with a medium effect. Chronic sleep problems (CSRQ) did not significantly change at any measurement occasion, but a small decrease in chronic sleep problems approached significance at 2-month follow-up. Children reported a small increase of emotional well-being (WHO-5) at posttest and 2-month follow-up, but this only approached significance. Emotional well-being was significantly increased at 1-year follow-up with a medium effect size. In addition to multilevel analyses, we assessed changes in the clinically relevant cutoff values on the CBCL and YSR. A large drop was found for scores above the subclinical cutoff value. At pre-test respectively 78, 47, and 57% of children scored above the subclinical cutoff values for internalizing, externalizing, and attention problems, while at 1-year follow-up, this was reduced to 42, 16, and 18%. For details, see Table 4.

**Table 4:** Amount of children with (sub)clinical levels of symptoms at each measurement occasion on Youth Self-Report (YSR), Child Behavior Checklist (CBCL) mother-report, or CBCL father-report before and directly after MYmind, at 2-month follow-up, and at 1-year follow-up

	Internalizing symptoms	Externalizing symptoms	Attention problems
Pre-test	35 (78%)	21 (47%)	26 (57%)
Posttest	28 (62%)	16 (35%)	18 (40%)
2-month follow-up	19 (42%)	9 (20%)	8 (18%)
1-year follow-up	19 (42%)	7 (16%)	8 (18%)

Subclinical level: T score ≥ 60 for internalizing and externalizing symptoms; T score ≥ 65 for attention problems

#### Mindful Awareness

Children reported no significant increase of mindful awareness (CAMM) from pre-test to posttest, 2-month follow-up, and 1-year follow-up.

### Parents’ Outcomes

#### Social Communication Problems

At posttest, a small significant reduction was found in social communication problems of parents (SRS-A). Social communication problems of parents did not significantly decrease at 2-month and 1-year follow-up.

#### Emotional and Behavioral Functioning

Parents reported a significant reduction with a small effect in their internalizing problems (ASR) at posttest. This reduction was significant with an approaching medium effect size at 2-month follow-up, and with a small effect at 1-year follow-up. Parents’ externalizing problems were also decreased at posttest with a small effect, at 2-month follow-up with a medium effect, and at 1-year follow-up with a small effect. Parents’ attention problems were significantly reduced at posttest and 2-month follow-up with a small effect. This reduction was not present at 1-year follow-up. Perceived stress of parents (PSS) was reduced at posttest and 2-month follow-up with a small effect. At 1-year follow-up, the decrease in stress was no longer significant.

#### Parenting

Parents’ stress about their competence in parenting (PSI-C) significantly decreased at posttest, 2-month, and 1-year follow-up with a small effect size. Parents reported a decrease in overreactivity in parenting (PS-O) with medium effect at posttest and 2-month follow-up. This effect remained significant with a small effect size at 1-year follow-up.

#### Mindful Awareness

**Fig. 3:**
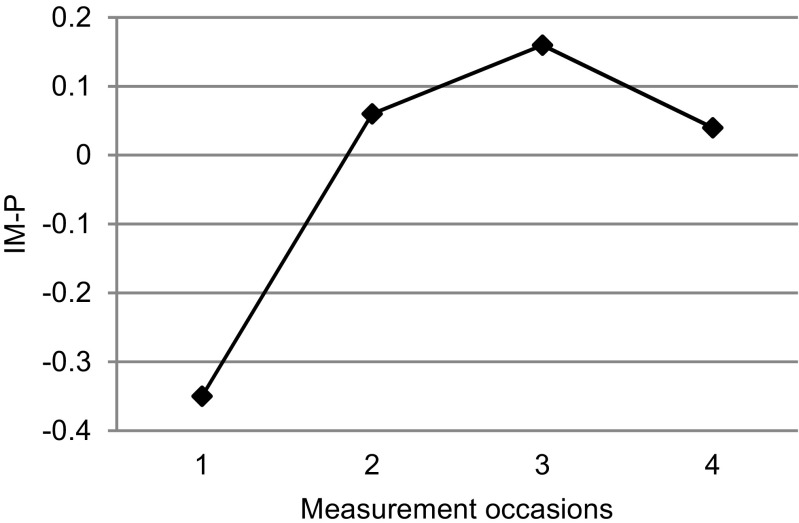
Mindful parenting of parents over time, as reported by parents on the Interpersonal Mindfulness in Parenting scale (IM-P). Standardized estimated marginal means are calculated based on the parameter estimates that were obtained through the multilevel analysis. *1* pre-test; *2* posttest; *3* 2-month follow-up; *4* 1-year follow-up

Parents reported a significant increase of mindfulness in parenting (IM-P) at posttest with a small effect, at 2-month follow-up with a medium effect, and at 1-year follow-up with a small effect (Fig. 3). Parent’s self-compassion (SCS-SF) significantly increased at posttest, 2-month, and 1-year follow-up with a small effect size.

### Predictors Interacting with Effects over Time

We explored whether differences over time could be explained by children’s age, attendance rate, or additional therapy. Main and interaction effects of these three variables are only described when significant. Attendance rate, calculated by summing the amount of sessions that a child and the parents were present (max. 27 times if both parents participated, excluding the booster session), was on average 19.22 (SD 5.16).

#### Children’s Age

A significant main effect of age was found on children’s rumination (parameter estimate = 0.36, *p* = .000), sleep problems (parameter estimate = 0.23, *p* = .000), emotional well-being (parameter estimate = −0.20, *p* = .001), and parent reported internalizing symptoms (parameter estimate = 0.10, *p* = .049). This indicates that older children reported higher levels of rumination and sleep problems, and lower levels of emotional well-being, and their parents reported higher levels of internalizing symptoms, across measurement occasions. In addition, a significant interaction effect between age and 2-month follow-up was found on sleep problems (parameter estimate = − 0.14, *p* = .022), indicating that older children reported more decrease in sleep problems than younger children.

#### Attendance Rate

A significant interaction effect between attendance rate and 2-month follow-up was found on children’s internalizing symptoms as reported by parents (parameter estimate = 0.05, *p* = .041). This indicates that higher attendance rate was related to less decrease in children’s internalizing symptoms. In addition, a significant interaction effect between attendance rate and 1-year follow-up was found on parents’ stress about their competence (parameter estimate = − 0.05, *p* = .031). This indicates that a higher attendance rate was related to more decrease in parents’ stress about their competence in parenting.

#### Additional Therapy

A significant interaction effect between additional therapy and posttest was found on parents’ overreactivity (parameter estimate = − 0.49, *p* = .034). A significant interaction effect between additional therapy and 1-year follow-up was found on parents’ internalizing symptoms (parameter estimate = − 0.55, *p* = .013) and on parents’ stress about their competence (parameter estimate = − 0.61, *p* = .008). This indicates that parents of families who received additional therapy, compared to parents of families who did not, reported more decrease in parental overreactivity at posttest, and more decrease in parental internalizing symptoms and stress about their competence in parenting at 1-year follow-up.

### Qualitative Results

Three main themes emerged from the conventional content analysis of children’s and parents’ experienced changes: *Mindfulness skills* included the aspects of mindfulness that participants learned. The two most frequently occurring subcategories were awareness and applying meditation. Four other subcategories were acceptance, pause before acting, concentration, and decentering. *Improved well-being* included the changes experienced as a consequence of practicing mindfulness. The three most frequently occurring subcategories were improved parent-child interaction, calmness, and coping with difficult experiences. Six other subcategories were decreased worry and rumination, decreased sleep problems, improved social interaction, improved mood, improved school-functioning, and other improvement. *Little to no change* included quotes of participants that experienced little to no change and was relatively small compared to the other main themes. For a description and example quotes of the subcategories, see Table 5.

**Table 5:** Description of main themes and subcategories of children’s and parents’ experienced change after Mymind

Main theme	Subcategories
Mindfulness skills	*Awareness*: Increased awareness in daily life, of the body, thoughts, or feelings, e.g., “living more conscious, in the here and now.”
	*Applying meditation*: Learned to apply meditation practices and increased knowledge about mindfulness, e.g., “more insight in the way mindfulness works.”
	*Acceptance*: Viewed themselves or others with greater compassion or acceptance, e.g., “less angry towards myself.”
	*Pause before acting*: Learned to concentrate or pause before acting.
	*Decentering*: Ability to distance themselves from feelings, thoughts, or stressful situations, e.g., “distancing from emotions.”
	*Concentration*: Better concentration and focus of attention.
Improved well-being	*Improved parent-child interaction*: Increased attention and calmness in parent-child interaction, improved relationship, e.g., by being more open and “better understand each other.”
	*Calmness*: Increased calmness and decreased stress, e.g., “‘I’m much calmer.”
	*Coping with difficult experiences*: Responding more calmly, for example “reacting less quickly,” increased coping with own feelings, e.g., “control them more effectively.”
	*Decreased worry and rumination*: Decrease in worrying and thinking, e.g., “about me feeling bad.”
	*Improved social interaction*: Better listening or “more social.”
	*Decreased sleep problems*: Sleeping better or more easy.
	*Improved mood*: A more balanced or less negative mood, e.g., “Now I am way less depressed.”
	*Improved school functioning*: e.g., “I am doing great at school.”
	*Other improvement*: a few quotes could not be categorized in one of the subcategories, e.g., “I can set priorities.”
Little to no change	e.g., “Little,” “Don’t know,” or “Nothing really.”

## Discussion

In this study, we investigated the direct, middle-term, and long-term effects of a mindfulness-based program with parallel sessions for children with ASD and their parents, MYmind, on quantitative and qualitative outcomes. Quantitative assessments took place along four outcome domains: social communication problems, emotional and behavioral functioning, parenting, and mindful awareness. For each of these domains, the results are discussed in relation to the qualitative results, previous studies, and possible explanations.

### Social Communication Problems

Children’s social communication problems decreased directly after MYmind, and this decrease lasted 2 months and 1 year after the intervention. This was supported by improved social interaction emerging as one of the experienced changes from the qualitative analysis. Also, it could be related to the experienced improvement in parent-child interaction, since parents reported about their child’s social communication problems, and they might report less problems if they experience improved interaction with their child. A decrease in adolescents’ social communication problems after MYmind was also found by De Bruin et al. (2015). Extending the previous pilot study, this study included younger children, from 8 years onwards. Since age did not affect changes over time, MYmind seems beneficial for this younger age group as well. In addition, this study shows that the decrease in social communication problems of children with ASD lasts up to 1 year later, a noteworthy finding considering that these social communication problems are chronically impaired in ASD (Seltzer et al. 2004). The theorized benefits of mindfulness for social interaction (Block-Lerner et al. 2007) seem to apply to children with ASD. Since neurocognitive deficits are theorized to be underlying the ASD symptomatology, the decreased social communication problems after the mindfulness-based program supports the suggestion that neurocognitive deficits in central coherence, executive functioning, and theory of mind could be improved by training mindfulness. In one of our future studies, we will investigate the effects of the MYmind program for children with ASD on neurocognitive functioning.

Parents’ social communication problems decreased directly after MYmind, but not on the middle- and long-term. Children, but not parents, were included based on their ASD symptoms, and parents scored on average within nonclinical ranges on the social responsiveness scale, so perhaps there was limited room for improvement in parents’ ASD specific social communication problems. Another explanation might be that the MYmind parent program focused on dealing with difficult parenting situations and parenting stress in relation to raising a child with ASD, rather than parents’ ASD specific social communication problems with other adults. The questionnaire that indexed social communication problems (SRS-A) mainly focusses on ASD specific social communication problems in interaction with other adults, rather than parent-child interactions (Constantino and Gruber 2005). Parents, however, did mention improved social interaction and parent-child interaction according to the qualitative analysis, such as listening with awareness and making issues better negotiable, indicating that parents did experience improved social interaction that was not captured in the standardized questionnaire.

### Emotional and Behavioral Functioning

Children’s emotional and behavioral functioning improved, including their internalizing, externalizing, attention problems, rumination, stress, and emotional well-being. This was also reflected in the large drop in percentages of children scoring above the clinically relevant cutoff value. Effects were most substantial 2 months after MYmind according to children, and were maintained up to 1 year later for externalizing problems, attention problems, and emotional well-being. Interestingly, directly after the training, children reported only decreased rumination and not yet improvement on other indicators of emotional and behavioral functioning, while according to parent-report, all child improvements were already present. It could be that the children first became *more* aware of their emotional and behavioral problems before they realized improvement, and therefore reported no improvement on most outcomes directly after MYmind, but only 2 months later. In mindfulness-based programs, participants train to become more aware of their internal experiences, including their thoughts, emotions, and behavior tendencies. This may lead to an increased report of emotional and behavioral dysfunction in the early stages of practicing mindfulness, because participants first become more aware of how chaotic their minds are before they can notice benefits (Davidson and Kaszniak 2015). According to the Learning Stages model (Burch 1970s, as in Adams 2016), children may have started the mindfulness training unconsciously incompetent with respect to their emotional and behavioral functioning. They then became consciously incompetent directly after the intervention, that is, they became aware of their emotional and behavioral difficulties. However, they could only apply what they learned in the mindfulness training to their emotional and behavioral problems in a consciously competent manner after more time had passed. Thereby, it could have helped that children were encouraged to continue practicing mindfulness after the final session of the MYmind program in a way that was tailored to their own needs and preferences, by making their personal meditation plan for the 2 months between posttest and follow-up. The improvements in children’s emotional and behavioral functioning strengthen the findings of previous smaller scale studies in which children’s rumination, aggressive behavior, and emotional and behavioral problems reduced (De Bruin et al. 2015; Hwang et al. 2015; Singh et al. 2011a, b).

Parents reported improvement in their own emotional and behavioral functioning as well, with internalizing and externalizing problems still decreased up to 1 year after the MYmind program. However, decreased attention problems and stress remained only until 2 months after the training. These findings strengthen the previous studies of mindfulness-based programs for parents of children with ASD that showed improved parental mental health (Dykens et al. 2014; Ferraioli and Harris 2013; Neece 2014). Furthermore, the present findings extend these previous studies in showing that benefits partly last up to 1 year later.

The improvements in children’s and parents’ emotional and behavioral functioning are further supported by improved well-being as one of the main experienced changes rising from the qualitative analysis, including increased calmness, decreased worry and rumination, and improved mood. A decrease in sleep problems was also mentioned as experienced change, but this decrease was not reflected in the quantitative measure for the children. It could be that only some children experienced a decrease in sleep problems, but not enough children experienced this decrease to be present in the quantitative measure. Or, it could be that only some children had sleep problems and therefore only some needed to improve on this outcome. Another explanation could be that the questionnaire for indexing sleep problems was not appropriate for this population. This questionnaire mainly focuses on the consequences of chronic sleep reduction, such as irritation and energy loss during day time, rather than the quality of sleep itself (Meijer 2008). However, children with ASD often experience irritation and energy loss irrespective from sleep quality. Therefore, the questionnaire differed from the qualitative reports of decreased sleep problems.

Interestingly, coping with difficult experiences came up as an important experienced change in the qualitative analysis. Parents and children were better able to respond calmly and with patience to difficult situations, and better able to cope with emotions. This may be the consequence of skills practiced during the mindfulness training, such as relating differently to experiences, viewing them as passing events, non-judgmentally. Thereby, participants practice to get less caught up in difficult thoughts, feelings, and situations. Also, letting go and responding with awareness rather than impulsively are skills practiced during the mindfulness training (Bögels and Restifo 2014; Kabat-Zinn 1994; Segal et al. 2012) that could have strengthened parents’ and children’s coping abilities. In addition, children and parents learned specific tools, such as the 3-min breathing space, to decrease their experienced stress in difficult situations. Thereby, they could have improved their emotional and behavioral functioning. Taking this together, a possible mechanism of change of the MYmind program is that the coping abilities of children with ASD and their parents increase, and thus become more in balance with their experienced demands. This in turn reduces psychological stress responses, such as their emotional and behavioral problems (Lazarus and Folkman 1984). This is further supported by the decrease in perceived stress by children and parents. So children were probably better able to cope with the high demands due to their ASD symptoms and underlying neurocognitive problems, and parents were probably better able to cope with the demanding situation of raising a child with ASD, leading to improved emotional and behavioral functioning. Another possible mechanism of change is that practicing mindfulness leads to a lower general stress level, as reflected in decreased stress and increased calmness on both qualitative and quantitative measures. Through a lower baseline stress level, children and parents may experience less frequent or less intense outbursts in stress responses to difficult situations, leading to improved emotional and behavioral functioning.

### Parenting

Parental overreactivity and stress about parents’ competence in parenting were decreased directly after the training, and this decrease remained 2 months and 1 year later. This was further supported by improved parent-child interaction as one of the main experienced improvements in well-being. Interestingly, in the previous pilot study by De Bruin et al. (2015), no decrease in parental overreactivity was found, while other parenting styles did improve. This discrepancy might be explained by the inclusion of younger children in the present study. Decrease in parenting stress is previously shown by multiple studies investigating mindfulness-based programs for parents of children with ASD (De Bruin et al. 2015; Dykens et al. 2014; Ferraioli and Harris 2013; Neece 2014). This study adds to the previous findings by showing that decreased parenting stress lasts up to at least 1 year after a mindfulness-based program. Parenting stress is reciprocally related to children’s emotional and behavioral problems (Bauminger et al. 2010; Hastings 2002). In the current study, children’s emotional and behavioral problems, parenting stress, and parenting behavior are improved on all measurement occasions, and with the current study design, we cannot test how these changes influence each other. That is, reduced parenting stress may improve child behavior, or improved child behavior may reduce parenting stress, or both. However, by combining a mindfulness-based program for children with ASD with mindful parenting training for their parents, all these aspects are targeted at the same time. Given the reciprocal relationship between parental stress and children’s emotional and behavioral problems in ASD (Bauminger et al. 2010; Hastings 2002), the combination may be reinforcing and therefore most beneficial for these families.

Notably, father involvement in the MYmind parent program was relatively large in this study; for more than 60% of the children, their father participated. Both parents were asked to participate in the training, but only one participating parent was required, and fathers were not specifically asked to participate. Since males are relatively more vulnerable to the genetic susceptibility for autistic traits and show increased autistic traits as compared to females (Bishop et al. 2004; Constantino and Todd 2003), a large father participation might reflect self-selection for the parent program to cope with their own social communication problems. Another explanation could be that parents decided to increase parenting involvement of fathers by participating in MYmind together with their child, because fathers report to be less stressed but also less involved in parenting children with ASD than mothers (Tehee et al. 2009). So, fathers may participate more often in MYmind for children with ASD, for their own benefit, to increase their involvement in parenting, and to lower the parenting burden for mothers.

### Mindful Awareness

In addition to outcome measures of social, emotional, behavioral functioning, and parenting, in this study, mindfulness process measures were used. Although this may help to investigate the specificity of the intervention, many studies in the research field of mindfulness-based programs for children lack mindfulness process measures (Tan 2016). Surprisingly, in the present study, children did not report significant changes in mindful awareness, while mindfulness skills was one of the main themes evaluated as experienced change in the qualitative analysis. Similar to the results of this study, no changes in adolescents’ mindful awareness were found in the previous pilot study into MYmind (De Bruin et al. 2015). Some studies into mindfulness-based programs for children and their parents did find an increase in adolescents’ mindful awareness (e.g., Bögels et al. 2008), but yet other studies show no effects on mindful awareness for adolescents (e.g., Haydicky et al. 2015; Van de Weijer-Bergsma et al. 2012). Apparently, the experienced change that emerged from the qualitative analysis was not reflected in the standardized questionnaire on mindful awareness. On the one hand, the lack of improvement in mindful awareness implies that the MYmind program does not improve children’s outcomes by training them in mindfulness. The intervention might work through other mechanisms, for example through sharing experiences with other children with ASD in this group setting, through increased mindful parenting, or through relaxation and decreased stress, since increased calmness emerged as an important experienced change. On the other hand, the measurement of mindfulness is conceptually and methodologically difficult (Davidson and Kaszniak 2015). Standardized questionnaires to index mindfulness are considered weak because of several reasons. For example, there is no possibility to use external referents or gold-standard measures to assess the validity, so whether questionnaires indeed measure mindfulness is uncertain. In addition, interpretation of the items depends on the experience an individual has with practicing mindfulness, and therefore children interpret the items differently after a mindfulness-based program. Also, mindfulness is conceptualized as an active practice or process, a state that changes moment-by-moment, which could be better assessed by its assumed psychological outcomes than by a standardized trait measures (Grossman 2011). Concluding, the lack of improvement on the standardized questionnaire of mindful awareness could reflect the weaknesses of measuring mindfulness in this way.

Parents did report an increase in mindful awareness, as assessed by mindful parenting and self-compassion, directly and on the middle- and long-term. This is further supported by mindfulness skills emerging as one of the main experienced changes in the qualitative analysis. An improvement in mindful parenting was also found in the previous pilot study into MYmind (De Bruin et al. 2015). Another mindfulness-based program for parents of children with ASD increased parental mindful awareness as well (Ferraioli and Harris 2013). The improvement in parental mindfulness suggests that the MYmind parent program reached its aims. Parents seem to have learned important aspects of a mindfulness-based program. Concentration, awareness of thoughts, feelings, and bodily sensations, present-moment awareness, decentering, acceptance, and letting go are described as what is to be learned in mindfulness-based programs (Segal et al. 2012), and these aspects were almost all described by parents as mindfulness skills they learned. In addition, parents seem to have learned to respond calmly instead of reacting automatically, a core aspect of the Mindful Parenting program (Bögels and Restifo 2014), as reflected by their experienced change to pause before acting and cope with difficult experiences by reacting more calmly. Since higher levels of mindful parenting, acceptance, and self-compassion seem to reduce the impact of children’s behavior problems on parental anxiety, depression, and stress (Jones et al. 2014; Neff and Faso 2015; Weiss et al. 2012), the increase in mindful parenting and parental self-compassion might be important mechanisms of change by which parents’ emotional and behavioral functioning were improved. Furthermore, an improvement in mindful parenting after a mindful parenting training predicts a decrease in children’s emotional and behavioral problems (Meppelink et al. 2016), thus parents improvement in mindful parenting could have benefited the children’s outcomes as well.

### Limitations, Strengths, and Future Research Recommendations

The results should be interpreted and generalized with caution for several reasons. Firstly, this study included repeated measures over time without a control intervention. Therefore, effects of MYmind cannot be distinguished from effects of time or child maturation. Secondly, part of the participants received additional therapy, which included a variety of treatment approaches and frequencies. When exploring whether additional therapy influenced effects over time, we found that families who received additional therapy reported more decrease in parental internalizing symptoms and in parental overreactivity at only one out of three measurement occasions, while no stronger benefits for these families were found on the other outcomes at any measurement occasion. Still, we cannot completely rule out that effects of the mindfulness-based program are influenced by effects of additional therapy. Finally, not all participants completed the questionnaires on all measurement occasions. This could influence the results in two ways. On the one hand, participants who did not complete the posttest and follow-up are more likely to perceive less benefits from the training and thereby less willing to participate in the study assessments. On the other hand, the power to detect significant improvement while improvement is present is decreased by missing data.

This study also has several strengths. We included a 1-year follow-up to assess whether effects last over the long-term. In addition, we used multi-informant data to assess children’s outcomes, providing stronger evidence for the results. Also, we did not only look at the quantitative results but also incorporated a qualitative analysis of subjective experienced change as well. This provides greater insights into the psychological aspects of practicing mindfulness (Grossman 2011). Furthermore, we developed a protocol to evaluate the treatment integrity of the investigated program and evaluated this study’s treatment integrity. Evaluating treatment integrity is an important quality in psychological intervention research and so far has received little attention in studies of mindfulness-based programs for children and parents (Harnett and Dawe 2012).

Before widespread implementation of this mindfulness-based program in clinical practice is recommended, we recommend future studies using randomized controlled trials to compare the effectiveness of a mindfulness-based program to an active control treatment, preferably in a group setting with the same time investment. Also, further exploration of the qualitative accounts of children’s and parents’ experiences can be a valuable contribution to standardized questionnaires. A qualitative approach provides helpful insights into personal experiences and attributions to change (Grossman 2011), which is particularly interesting given the heterogeneity of people with ASD. Moreover, studies are needed to compare the effects of MYmind in an individual versus a group format, as it is not yet clear whether children with ASD benefit best in a group or individual family setting. In addition, future studies could investigate the mechanisms of change of mindfulness-based programs for children with ASD and their parents, such as decreased neurocognitive deficits, improved coping abilities, decreased stress, or increased mindful parenting.

## Conclusion

Children, including adolescents, with ASD and their parents could benefit from MYmind, as results show improvements in social communication problems, emotional and behavioral functioning, parenting, and parental mindful awareness, which seem to last up to 1 year after the intervention. Importantly, this implies that a mindfulness-based program can improve both ASD symptoms and common comorbid emotional and behavioral problems for children with a wide variety of ages, and could support families in coping with the demanding consequences of ASD.
